# Intermedin Stabilized Endothelial Barrier Function and Attenuated Ventilator-induced Lung Injury in Mice

**DOI:** 10.1371/journal.pone.0035832

**Published:** 2012-05-01

**Authors:** Holger Christian Müller-Redetzky, Wolfgang Kummer, Uwe Pfeil, Katharina Hellwig, Daniel Will, Renate Paddenberg, Christoph Tabeling, Stefan Hippenstiel, Norbert Suttorp, Martin Witzenrath

**Affiliations:** 1 Department of Infectious Diseases and Pulmonary Medicine, Charité-Universitätsmedizin Berlin, Germany; 2 Institute for Anatomy and Cell Biology, Universities of Giessen and Marburg Lung Center, Justus-Liebig-University Giessen, Germany; Louisiana State University, United States of America

## Abstract

**Background:**

Even protective ventilation may aggravate or induce lung failure, particularly in preinjured lungs. Thus, new adjuvant pharmacologic strategies are needed to minimize ventilator-induced lung injury (VILI). Intermedin/Adrenomedullin-2 (IMD) stabilized pulmonary endothelial barrier function in vitro. We hypothesized that IMD may attenuate VILI-associated lung permeability in vivo.

**Methodology/Principal Findings:**

Human pulmonary microvascular endothelial cell (HPMVEC) monolayers were incubated with IMD, and transcellular electrical resistance was measured to quantify endothelial barrier function. Expression and localization of endogenous pulmonary IMD, and its receptor complexes composed of calcitonin receptor-like receptor (CRLR) and receptor activity-modifying proteins (RAMPs) 1–3 were analyzed by qRT-PCR and immunofluorescence in non ventilated mouse lungs and in lungs ventilated for 6 h. In untreated and IMD treated mice, lung permeability, pulmonary leukocyte recruitment and cytokine levels were assessed after mechanical ventilation. Further, the impact of IMD on pulmonary vasoconstriction was investigated in precision cut lung slices (PCLS) and in isolated perfused and ventilated mouse lungs. IMD stabilized endothelial barrier function in HPMVECs. Mechanical ventilation reduced the expression of RAMP3, but not of IMD, CRLR, and RAMP1 and 2. Mechanical ventilation induced lung hyperpermeability, which was ameliorated by IMD treatment. Oxygenation was not improved by IMD, which may be attributed to impaired hypoxic vasoconstriction due to IMD treatment. IMD had minor impact on pulmonary leukocyte recruitment and did not reduce cytokine levels in VILI.

**Conclusions/Significance:**

IMD may possibly provide a new approach to attenuate VILI.

## Introduction

Mechanical ventilation (MV) is a life saving treatment without alternatives in acute respiratory failure, and MV is also employed following surgery or trauma. One third of all patients in intensive care units are receiving MV [1]. Notably, even a minimum of physical forces on lung tissue induced by MV may evoke ventilator-induced lung injury (VILI), an important undesirable effect of respirator therapy [2]. Minimization of MV-induced physical stress by reduction of tidal volumes to 6 ml/kg significantly improved clinical outcome of mechanically ventilated patients [3]. However, particularly preinjured lungs are sensitive for the development of VILI even in the setting of lung-protective ventilation [4], [5]. VILI is characterized by pulmonary inflammation with liberation of cytokines, recruitment of leukocytes to the lung and particularly increased lung permeability, resulting in lung edema, surfactant dysfunction, impaired lung compliance and deterioration of pulmonary gas exchange [6]. As the necessity to guarantee sufficient gas exchange frequently limits a further substantial reduction of tidal volumes and oxygen supply, new adjuvant pharmacological therapies enhancing pulmonary vascular barrier function in VILI/acute respiratory distress syndrome (ARDS) in addition to lung-protective ventilation are needed to prevent or ameliorate VILI.

We have previously shown that the potent barrier protective properties of the calcitonin receptor-like receptor (CRLR) agonist adrenomedullin reduced VILI in different murine VILI models [7]. Intermedin (IMD), alternatively named adrenomedullin-2, is an endogenous peptide also signaling via CRLR coupled to the receptor activity-modifying proteins (RAMP) 2 or 3 [8], [9]. Intermedin stabilized endothelial barrier function in vitro and in an isolated mouse lung model of hydrostatic lung edema [10], [11], and reduced pulmonary leukocyte infiltration in pulmonary ischemia/reperfusion injury [12]. Further, IMD had protective effects in cardiac ischemia/reperfusion injury and lowered systemic blood pressure [8], [13], [14].

Pulmonary hyperpermeability and hyperinflammation are hallmarks of VILI and ARDS, and pharmacologic attenuation of vascular barrier breakdown in these conditions may be a promising therapeutic approach to limit VILI in addition to lung protective ventilation strategies. We hypothesized that the CRLR ligand IMD has barrier protective effects in a murine model of VILI in vivo.

In the current study we observed IMD-evoked barrier stabilization on endothelial cell layers in vitro. In a clinically relevant mouse model of VILI, continuously infused IMD reduced VILI-induced pulmonary hyperpermeability without affecting pulmonary and systemic inflammation. IMD revealed vasodilatatory effects on pulmonary vasculature in mouse lungs. Further, the influence of MV on pulmonary IMD expression and distribution as well as on expression of the IMD receptor components was investigated.

**Figure 1:**
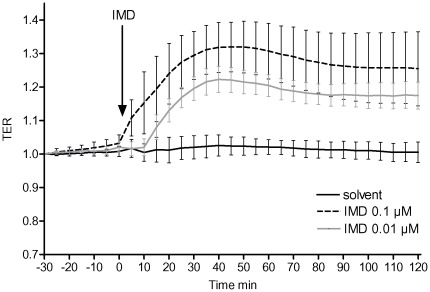
IMD improved endothelial barrier function in endothelial cells. HPMVECs grown to confluence on gold microelectrodes to measure transcellular electrical resistance (TER) were stimulated with 0.01 or 0.1 µM IMD or with solvent. IMD dose dependently improved endothelial barrier function as displayed by TER increase. (n = 6).

**Figure 2:**
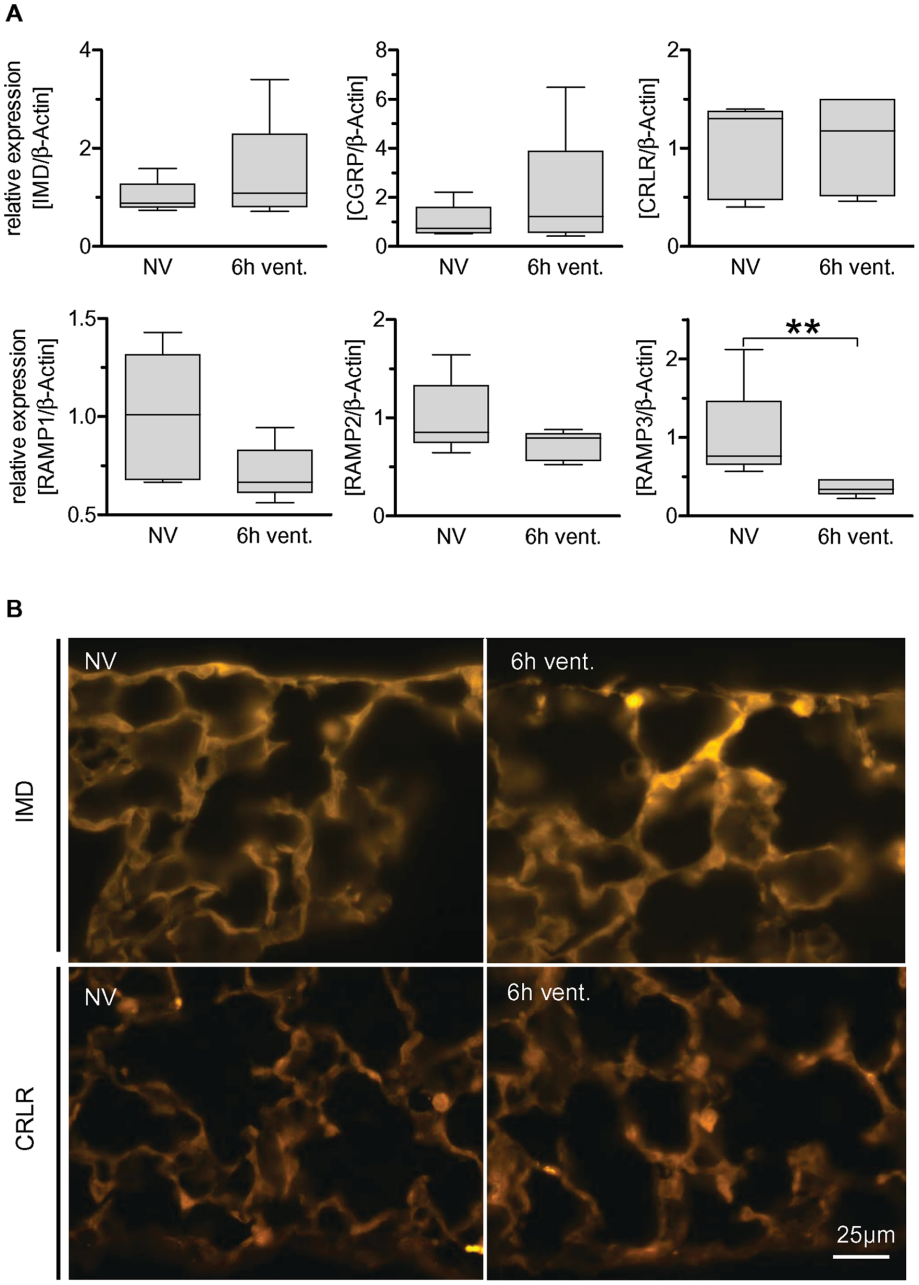
Regulation of IMD and its receptor complexes in VILI. Mice were ventilated with a tidal volume of 12 ml/kg for 6 h (6****h vent.). Non ventilated individuals (NV) served as controls. A) Regulation of IMD, CGRP and RAMP1-3 was quantified by qRT-PCR in the lungs of ventilated (6****h vent.) and non ventilated mice (NV). (** p<0.01, n = 5). B) Immunofluorescence analysis of subpleural peripheral lung. IMD-immunolabelling was slightly enhanced in ventilated (6****h vent.) compared to non-ventilated mice (NV) while CRLR-immunolabelling did not differ between groups. Tissue sections depicting NV and 6****h vent groups were processed simultaneously and images were taken at the same exposure time (30 ms for IMD and 150 ms for CRLR).

## Methods

### Ethics Statement

Animal experiments were approved by the animal ethics committee of the Charité-Universtätsmedizin Berlin and local governmental authorities (LAGeSo (Landesamt für Gesundheit und Soziales Berlin), approval ID: G 0100/10).

### Transcellular Electrical Resistance (TER) of Endothelial Cells

Human pulmonary microvascular endothelial cells (HPMVEC) were purchased from PromoCell (Heidelberg, Germany) and grown on evaporated gold electrodes, connected to an electrical cell-substrate impedance system (Applied Biophysics, Troy, NY, USA). Cells were exposed to 0.01 or 0.1 µM human IMD (1–47, Phoenix, Burlingame, CA, USA) or solvent and TER values from each microelectrode were continuously monitored as described previously [15].

### Mice

Female C57Bl/6 mice (8 to 10 weeks; 18 to 20 g; Charles River, Sulzfeld, Germany) were used.

### Mechanical Ventilation and IMD Treatment

As described previously [16] mice were anesthetized by intraperitoneal injections of Fentanyl (75 µg/kg), Midazolam (1.5 mg/kg) and Medetomedin (0.75 mg/kg). Repetitively, Fentanyl (16 µg/kg), Midazolam (0.33 mg/kg) and Medetomedin (0.16 mg/kg) was supplied via an intraperitoneal catheter when required to guarantee adequate anaesthesia during the observation period. Body temperature was maintained at 37°C by a temperature-controlled heating pad. After tracheotomy and intubation, mice were ventilated (MiniVent, Hugo-Sachs-Electronics, March-Hugstetten, Germany) with 70% oxygen; tidal volume (V_T_) 7 ml/kg; respiratory rate (RR) 240 minute^–1^; positive end-expiratory pressure (PEEP) 6****cmH_2_O. A carotid artery catheter was placed for blood pressure monitoring and infusion of NaCl 0.9% containing 100 mmol/l HCO^3–^ (350 µl/h). There was no additional fluid support in any conducted experiment. A urinary catheter was inserted. V_T_, RR, airway pressure, peripheral oxygen saturation and urine output were monitored (Pulmodyn, Hugo-Sachs-Electronics, March-Hugstetten, Germany; MouseOx, STARRLife-Sciences, Oakmont, PA, USA). After preparation, a recruitment manoeuvre was performed (airway pressure 35****cmH_2_O for 5 sec) before respirator settings were adjusted for 6 h to V_T_ 12 ml/kg, RR 120 min^–1^, PEEP 2****cmH_2_O. A second recruitment manoeuvre was performed 5 min before termination of the experiment. All mice survived the protocol. At termination of the experiments mice were sacrificed by exsanguination via the carotid artery catheter. Blood samples were analyzed for p_a_O_2_ and lactate by blood gas analyzer (ABL-800, Radiometer, Copenhagen, Denmark). P/F ratio was calculated as p_a_O_2_/FiO_2_. Non-ventilated mice served as controls. Murine IMD (1–47, Phoenix, Burlingame, CA, USA) (0.025 mg/kg/h) was continuously infused via the carotid artery catheter starting with onset of ventilation.

**Figure 3:**
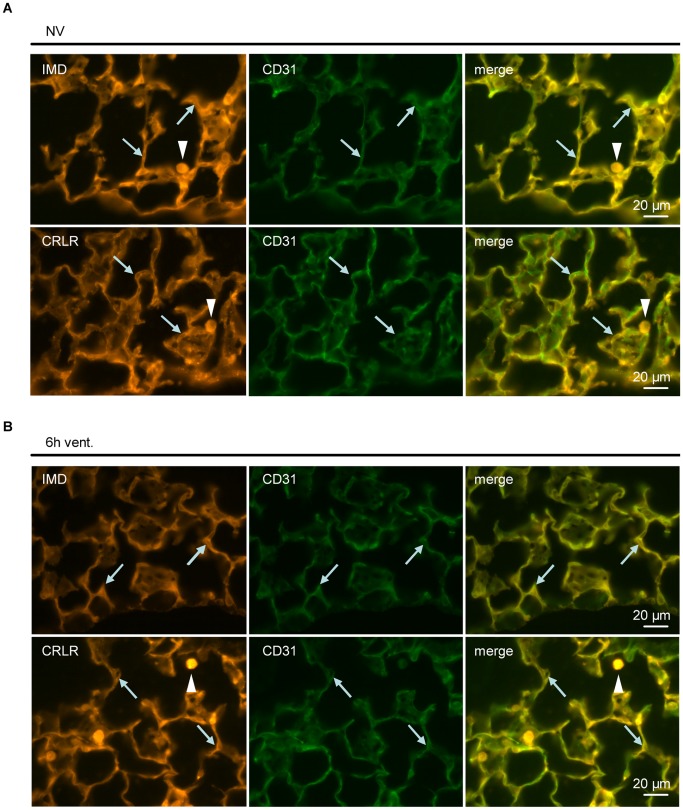
Pulmonary distribution of IMD and CRLR was not altered in VILI. In non ventilated mice (A) and individuals ventilated with a tidal volume of 12 ml/kg for 6 h (6****h vent.) (B) IMD- and CRLR-immunolabelling colocalized with CD31-immunoreactivity (*arrows*), a marker for endothelial cells. In addition, alveolar macrophages were IMD- and CRLR-positive (*arrowheads*). (n = 5, representative images shown).

**Figure 4:**
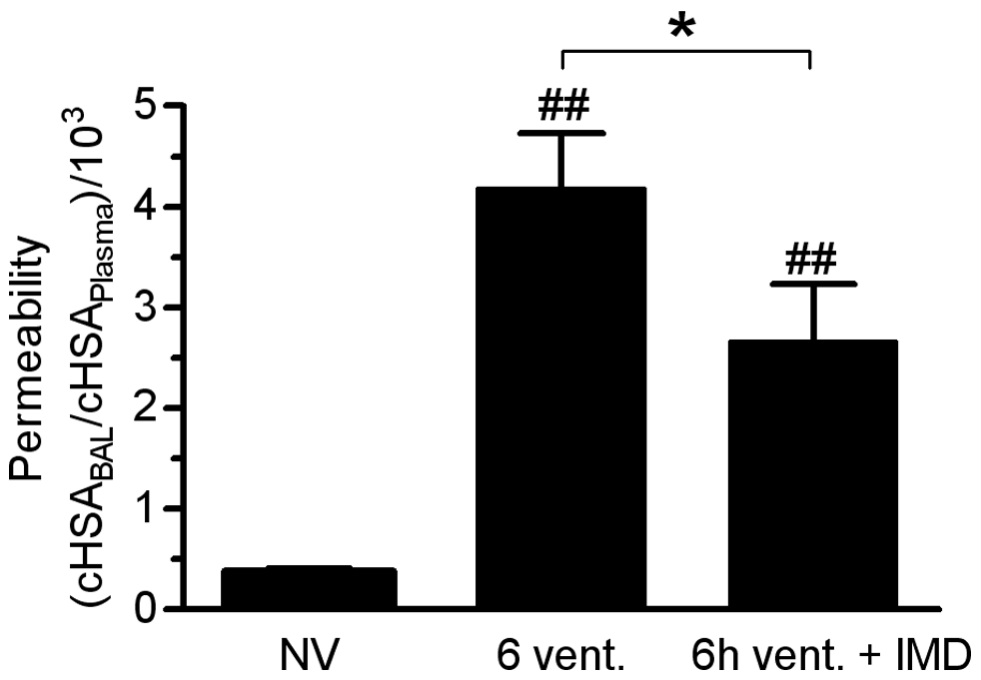
IMD reduced pulmonary vascular hyperpermeability in VILI. Mice were ventilated with a tidal volume of 12 ml/kg for 6 h and treated with IMD 0.025 mg/kg*h (6****h vent.+IMD) or solvent (6****h vent). NV  =  non ventilated mice. Human serum albumin (HSA) was infused 90 min prior to termination of the experiment. HSA concentration (cHSA) in plasma and BAL were determined. Increased HSA BAL/plasma ratio indicated microvascular leakage. (*p<0.05, ## p<0.01 vs. NV, NV n = 5, 6****h vent. n = 7, 6****h vent. + IMD n = 8)

### qRT-PCR

Lungs were flushed and snap frozen in liquid nitrogen. Total RNA was isolated from the lungs using RNeasy mini kit (Qiagen, Hilden, Germany) according to the manufacturer’s instructions. To remove genomic DNA contamination, isolated RNA samples were treated with 1 U DNase/µg RNA (Invitrogen, Karlsruhe, Germany) for 15 min at 37°C. One microgram of total RNA was used in a 20 µl reaction to synthesize cDNA using Superscript H^-^ reverse transcriptase (200 U/µg RNA, Invitrogen) and oligo dTs as primers. Reverse transcription reaction was for 50 min at 42°C. Real-time quantitative PCR was performed using the I-Cycler IQ detection system (Bio-Rad, Munich, Germany) in combination with the IQ SYBR Green Real-Time PCR Supermix (Bio-Rad). The PCR conditions included initial denaturation in one cycle of 10 min at 95°C followed by 40 cycles of 20 s at 95°C, 20 s at 60°C, and 20 s at 72°C. The relative expressions were calculated as: 2^-(ΔCT)^ × 1/mean control 2^-(ΔCT)^, where ΔCT is calculated as: ΔCT = CT_GOI_-CT_HKG_ (GOI: gene of interest, HKG: house keeping gene). Primer sequences are provided in Table S1.

### Immunofluorescent Staining

Immunolabelling for IMD and CRLR immunolabelling was performed by overnight incubation at room temperature with previously characterized antibodies [11], [17], including double-labelling with biotinylated rat monoclonal anti-CD31 (1 µg/ml; clone MEC 13.3, BD Biosciences, Heidelberg, Germany), an endothelial marker [18], and biotinylated lycopersicon esculentum (LEA) lectin (1∶3200; Vector Laboratories, Burlingame, CA, USA), a type I alveolar epithelial cell marker [19]. Secondary reagents, each applied for 1 h, were Cy3-conjugated goat anti-human IgG F(ab)_2_ (1∶500; Dianova, Hamburg, Germany), Cy3-conjugated donkey anti-rabbit IgG (1∶2000; Dianova), and FITC-conjugated streptavidin (1∶500; Sigma, Deisenhofen, Germany). Optionally, nuclei were counterstained by adding 0.4 µg/ml 4′,6-diamidino-2′-phenylindole-dihydrochloride (DAPI; Sigma) to the secondary reagent solution.

**Figure 5:**
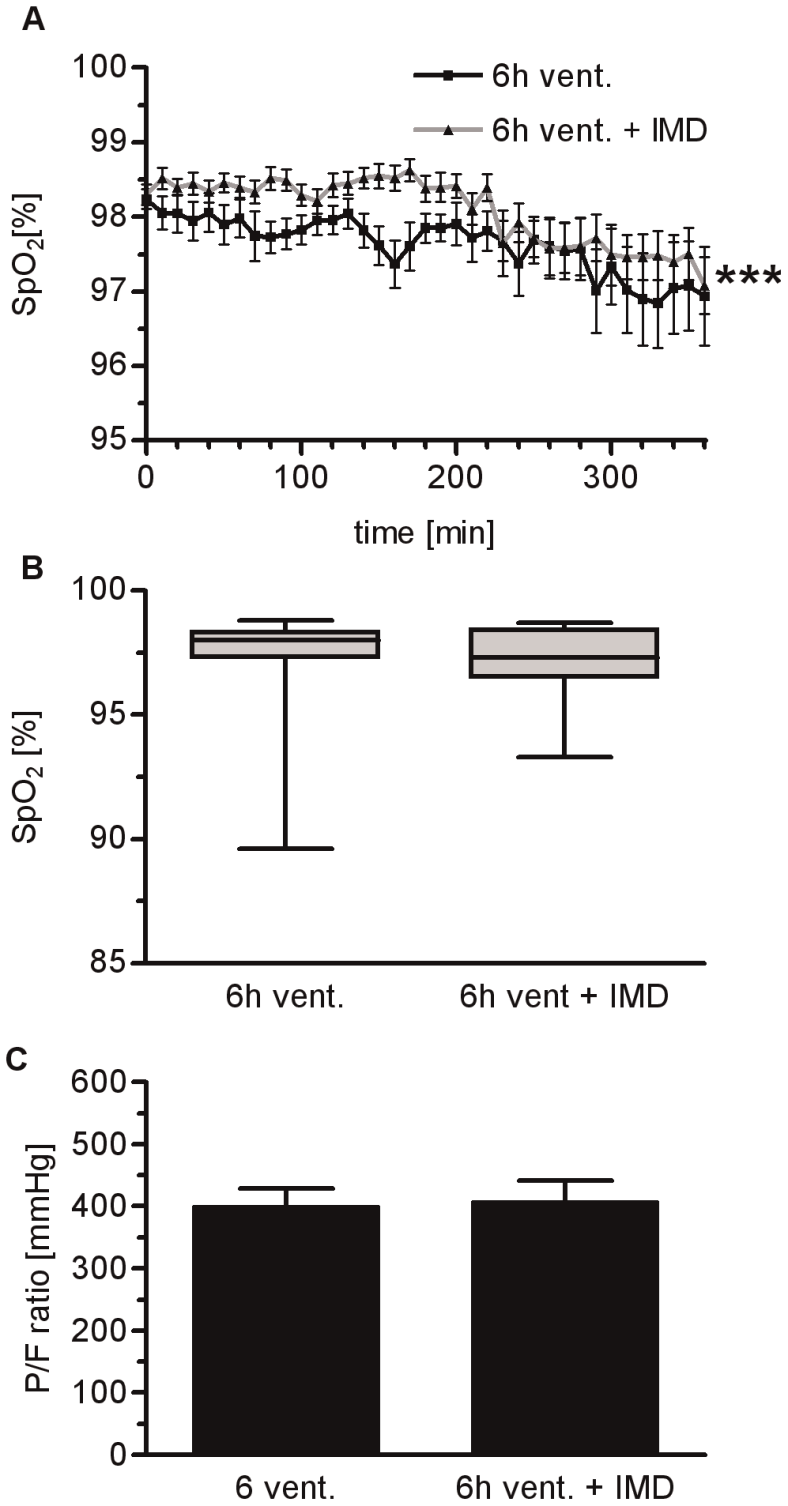
IMD had no impact on oxygenation in VILI. Mice were ventilated with a tidal volume of 12 ml/kg for 6 h and treated with IMD 0.025 mg/kg*h (6****h vent.+IMD) or solvent (6****h vent). A) Peripheral SpO_2_ was monitored. B) At the end of the 6****h ventilation period, SpO_2_ was not relevantly different between groups. C) After 6****h of MV, the P/F ratio was not different between groups. (***p<0.001, n = 15)

**Figure 6:**
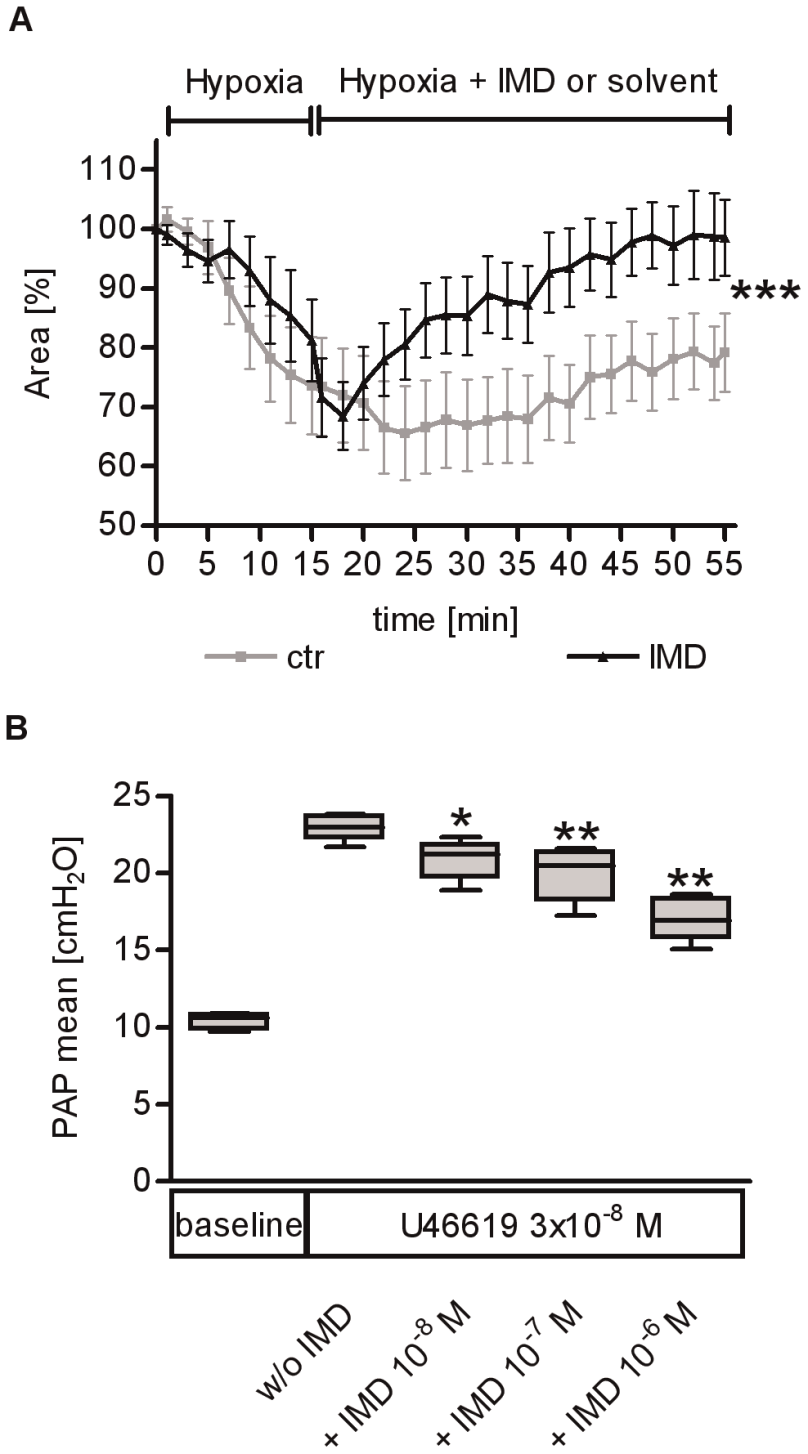
IMD mediated pulmonary vasodilation and reversed hypoxic pulmonary vasoconstriction. A) In precision cut lung slices (PCLS) hypoxic pulmonary vasoconstriction (HPV) was induced, and the luminal areas of single intra-acinar pulmonary arteries were continuously analyzed by planimetry. HPV was markedly reduced by 500 nM IMD (***p<0.001; n = 10). B) In isolated ventilated and perfused mouse lungs the thromboxane agonist U46619 induced a marked elevation of pulmonary artery pressure (PAP) during constant perfusion flow. PAP was lowered dose dependently by IMD bolus injection (* p<0.05, ** p<0.01, n = 5)

### Lung Permeability

At the end of the experiment BAL and plasma concentration of previously injected human serum albumin (cHSA) was determined and permeability was assessed by calculating the cHSA BAL/Plasma ratio as described previously [16].

**Figure 7:**
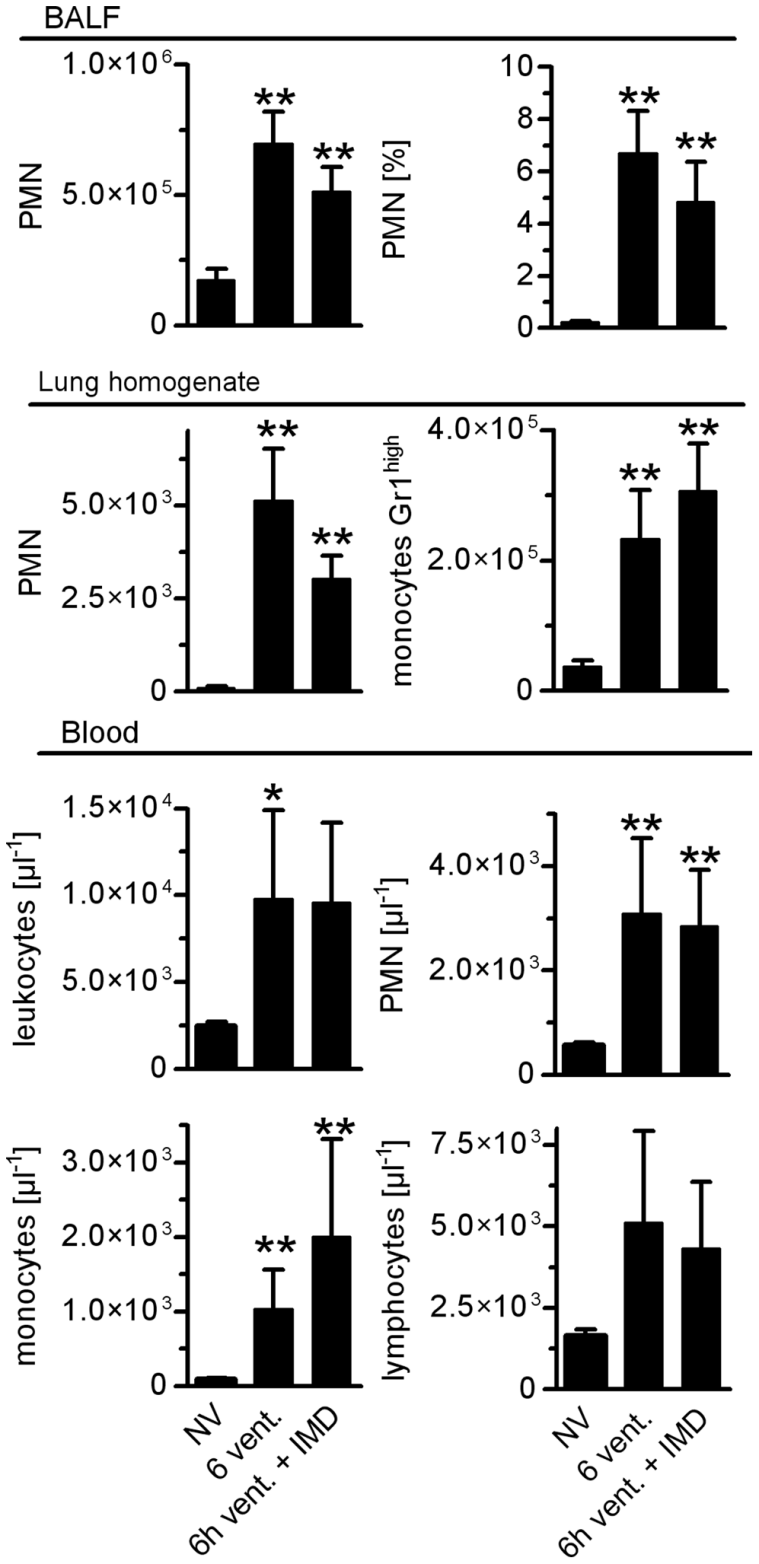
VILI induced pulmonary leukocyte recruitment and blood leukocytosis were unaffected by IMD treatment. Mice were ventilated with a tidal volume of 12 ml/kg for 6 h and treated with IMD 0.025 mg/kg*h (6****h vent.+IMD) or solvent (6****h vent). NV  =  non ventilated mice. Leukocytes isolated from bronchoalveolar lavage fluid (BALF), lung homogenate and blood, respectively, were quantified and differentiated by flow cytometry (*p<0.05 vs. NV, **p<0.01 vs. NV, NV n = 5, 6****h vent. n = 7, 6****h vent. + IMD n = 8)

**Figure 8:**
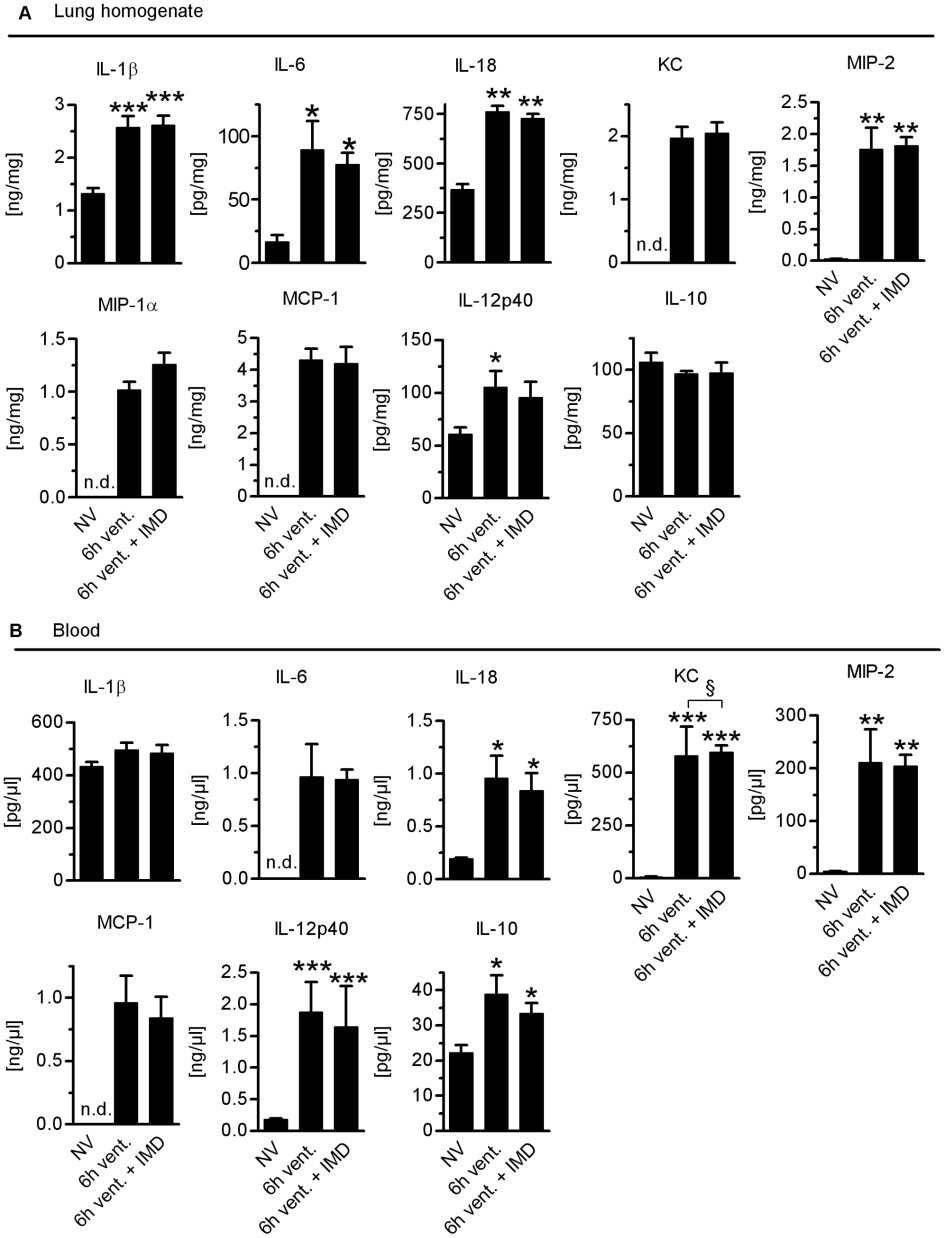
VILI-induced cytokine production was not altered by IMD. Mice were ventilated with a tidal volume of 12 ml/kg for 6 h and treated with IMD 0.025 mg/kg*h (6****h vent.+IMD) or solvent (6****h vent). NV = non-ventilated mice. A) IL-1β, IL-18, IL-6, KC, MIP-1α, MIP-2, MCP-1, IL12p40 were detemined in lung homogenate by multiplex assay technique. (*p<0.05 vs. NV, **p<0.01 vs. NV n = 8) B) IL-1β, IL-18, IL-6, KC, MIP-1α, MIP-2, MCP-1, IL12p40 were determined in plasma by multiplex assay technique. (*p<0.05 vs. NV, **p<0.01 vs. NV n = 8).

### Hypoxic Vasoconstriction in Precision Cut Lung Slices (PCLS)

PCLS were prepared as described previously [20], [21]. Briefly, mice were killed by cervical dislocation and the airways were filled with 1.5% low melting point agarose. After solidification of the agarose, the lungs were cut into 200 µm thick slices. The agarose was removed by incubation of the PCLS in phenolred-free minimal essential medium (MEM) continuously gassed with 21% O_2_, 5% CO_2_, 74% N_2_ for at least 2 h at 37°C. To analyze vasoreactivity of individual cross-sectioned intra-acinar arteries (minimal inner diameter up to 40 µm), PCLS were transferred into a flow-through superfusion chamber (Hugo Sachs Elektronik, March, Germany). At the beginning of each experiment the capability of the vessel to contract in response to the thromboxane analogue U46619 and to dilate after application of the NO donor sodium nitroprusside was checked. After washing out these drugs with normoxic gassed in phenolred-free MEM (21%O_2_, 5% CO_2_, 74% N_2_) PCLS were incubated with hypoxic gassed medium (1%O_2_, 5% CO_2_, 94% N_2_; 0.7 ml/min). After 15 min 500 nM of murine IMD was added to the hypoxic medium. Pictures of the artery were taken every 2 min using an inverted microscope mounted on the superfusion chamber. Changes of the luminal area of the vessels were evaluated by lining the inner boundaries by hand. The luminal area at the beginning of the experiment was defined as 100% and vasoreactivity was expressed as relative decrease or increase of this area. In the graph shown only the values obtained for the hypoxic incubation followed by the incubation in hypoxic medium +/- IMD are given. For a clear graphic presentation the values obtained at the beginning of hypoxia were defined as 100%.

### Isolated Perfused and Ventilated Mouse Lungs

Lungs were prepared as described [22], [23]. Briefly, anesthetized mice were tracheotomised and ventilated. After sternotomy and cannulation of left atrium and pulmonary artery, lungs were perfused with 37°C sterile Krebs-Henseleit hydroxyethylamylopectine buffer (1****mL/min) and ventilated by negative pressure (P_exp_ –4.5, P_ins_ –9.0 cm H_2_O) in a humidified chamber. After a steady state period of 30 min, the thromboxane receptor agonist U46619 (3 × 10^–8^ M) was administered to the perfusate, evoking an increase in pulmonary arterial pressure. Murine IMD was administered to the perfusate for 30 seconds in 12-min intervals with IMD concentrations being increased. Maximum pressure response after IMD administration was determined.

### Leukocytes in Lung Tissue, BAL Fluid and Blood

Lungs were flushed. The left lung was digested in RPMI containing collagenase and DNAse for 1 h. Leukocytes were extracted by meshing the lung tissue through a cell strainer (100 µm). BAL cells were isolated by centrifugation of BAL fluid gained as described above. Lung and BAL leukocytes were counted by haemocytometer and blood leukocytes were quantified using TruCount-Tubes. Leukocytes were differentiated by flow cytometry according to their sidescatter/forward-scatter properties and CD45, Gr-1 and F4-80 expression (FACSCalibur, BD, Heidelberg, Germany).

### Quantification of Cytokines

Cytokines were quantified from total protein of flushed homogenized left lungs and from plasma samples (BioRad, Hercules, CA, USA).

### Data Analyses

Data are expressed as mean ± SEM. For comparison between groups, Mann-Whitey U-test was used. For the comparison of continuously measured data between groups, 2-way ANOVA was applied. P values <0.05 were considered statistically significant.

## Results

### IMD Stabilized Endothelial Barrier Function in HPMVECs

Confluent monolayers of HPMVECs stimulated with 0.01 or 0.1 µM IMD showed a dose dependent increase in TER displaying improved endothelial barrier integrity. This effect lasted for the whole observation period of 120 min (Fig. 1). Comparable improvement of barrier function was observed in primary human umbilical vein endothelial cell monolayers (data not shown in detail).

### Pulmonary RAMP3 mRNA Levels were Down Regulated in VILI

The impact of mechanical ventilation on endogenous IMD and its receptors was determined prior to exogenous IMD application. The influence of mechanical ventilation on the mRNA expression of IMD and its receptor compounds CRLR, RAMP1-3 as well as on the CRLR/RAMP1 agonist calcitonin gene-related peptide (CGRP) was assessed by qRT-PCR. VILI caused down regulation of pulmonary RAMP3 mRNA levels while the mRNA levels of IMD, CRLR, CGRP and RAMP1-2 were not affected by MV (Fig. 2A). IMD and CRLR protein expression and distribution in lung tissue was not altered by MV.

To investigate the effect of VILI on pulmonary IMD and CRLR protein expression and distribution in the lungs, immunofluorescence analysis was performed. The IMD specificity of the employed antibody was validated in preabsorption experiments (Fig. S1). A trend towards increased pulmonary IMD expression in VILI was observed (Fig. 2B). CRLR expression was not altered by MV (Fig. 2B). IMD and CRLR protein were located mainly, although not exclusively, in alveolar macrophages and in the pulmonary endothelium, which was confirmed by double staining of IMD or CRLR and the endothelial marker CD31 (Fig. 3A). These expression patterns were not altered by MV (Fig. 3B). Co-labelling with LEA, a marker for type I alveolar epithelial cells, demonstrated limited colocalization. Instead, large epithelial stretches without IMD- or CRLR-immunolabelling were evident (Fig S2).

### Exogenous IMD Reduced Pulmonary Hyperpermeability in VILI

As we had been observing a robust barrier stabilizing effect of IMD in vitro, and after having characterized the impact of MV on the endogenous IMD system we aimed to investigate whether continuously infused IMD protects against VILI induced pulmonary hyperpermeability in mechanically ventilated mice. Translocation of human serum albumin (HSA) from the vascular into the alveolar compartment was quantified after 6****h of MV and served as a marker for lung permeability. MV provoked a marked increase of lung vascular permeability, thereby indicating VILI. Treatment with exogenous IMD significantly reduced VILI-evoked pulmonary hyperpermeability (Fig. 4). As increased permeability is the crucial mechanism for the development of high permeability lung edema in ALI/VILI, pharmacologic targeting of this major pathomechanism by IMD is promising.

### IMD Infusion Lowered Systemic Arterial Blood Pressure

IMD is a vasoactive peptide causing vasodilation in rodents. Arterial hypotension and consecutive disturbance of the microcirculation thus had to be excluded to avoid interference of shock with VILI and IMD treatment. Mean systemic arterial blood pressure (MAP) was measured continuously by means of a catheter in the carotid artery of each ventilated individual. Blood lactate was chosen as an indicator for disturbance of the microcirculation and urine output during the last two hours of the experiment served as an indicator for parenchymal perfusion pressure and renal function. IMD treatment resulted in a significant reduction of MAP. Notably, this decrease in blood pressure did not impact blood lactate levels and urinary output, suggesting that systemic microcirculation and renal perfusion were not relevantly compromised (Fig S3). In additional experiments, an increased IMD dose (0.075 mg/kg/h) resulted in blood lactate levels of 56 +/- 13 mg/dl (n = 4) indicating a significant deterioration of the microcirculation despite a constant mean arterial blood pressure between 60–65 mmHg throughout the experiment (data not shown). Blood lactate levels in the control group were 37 +/- 5 mg/dl respectively. Thus, the dose of 0.025 mg/kg/h IMD was chosen, which reduced hyperpermeability while severe side effects due to haemodynamic alterations were not evident.

### Peripheral Oxygen Saturation in VILI was Slightly Improved by IMD Treatment

Improvement of oxygenation is not necessarily associated with improved survival in ARDS. However, loss of oxygenation capacity is a key marker of lung injury, and improvement of oxygenation would provide evidence for the efficacy of IMD associated reduction of VILI. Therefore, peripheral oxygen saturation was measured continuously throughout the experiments by pulse oxymetry, and pO2 was determined in arterial blood by a routine blood gas analyzer.

IMD treated individuals showed significantly (p<0.001) but only slightly improved peripheral oxygen saturation as compared to untreated ventilated mice throughout the observation period (Fig. 5A). However, following a final recruitment manoeuvre there was no detectable difference between groups regarding SpO_2_ and P/F ratio (Fig. 5B; Fig. 5C).

### IMD Reduced Pulmonary Vasoconstriction

With regard to the missing improvement in oxygenation despite barrier-stabilizing properties of IMD, we hypothesized that vasodilatory properties known for IMD might counteract reduction of lung injury by an increase of ventilation/perfusion mismatch. Indeed, hypoxic pulmonary vasoconstriction (HPV) was reduced by IMD in murine precision cut lung slices of mice (Fig. 6A). Further, IMD dose dependently reduced pulmonary vasoconstriction evoked by the thromboxane receptor agonist U46619 in isolated ventilated and perfused mouse lungs (Fig. 6B). Thus, an IMD related increase of the ventilation/perfusion mismatch possibly overrode improvement of barrier function due to IMD treatment.

### IMD did not Reduce Inflammation in VILI

Activation of the innate immune system plays a key role in the pathophysiology of VILI, and dampening this response can ameliorate VILI. However, interference with pathways of the innate immune system may render ARDS patients even more susceptible towards infections and cause unfavorable outcomes. Thus, we aimed to determine the impact of IMD on crucial innate immune responses in VILI. We analyzed leukocyte counts in the BAL fluid, lung homogenate and blood and quantified cytokines in lungs and blood. VILI evoked recruitment of neutrophils and Gr-1high monocytes as detected in BAL fluid and in lung homogenates. IMD did not impact pulmonary recruitment of neutrophils or Gr-1high monocytes in VILI (Fig. 7). VILI provoked blood leukocytosis with elevated numbers of monocytes and neutrophils, which was not modified by IMD treatment (Fig. 7). VILI increased pulmonary levels of IL-1β, IL-18, IL-6, KC, MIP-1α, MIP-2, MCP-1 and IL12p40, while IL-10 levels remained at baseline. IMD treatment did not alter pulmonary cytokine profiles (Fig. 8A). Also, plasma levels of IL-18, IL-6, KC, MIP-2, MCP-1, IL-12p40 and IL-10 were increased in mice ventilated for 6 h. IL-1β levels remained at baseline. IMD did not alter levels of measured plasma cytokines (Fig. 8B). Notably, in additional experiments with a higher dosage of IMD (0.075 mg/kg/h) we observed a trend towards reduced neutrophil counts in the BAL fluid (p = 0.0571, n = 4) and reduced monocyte counts in blood (p = 0.0286 n = 4) due to IMD treatment. As outlined above, 0.075 mg/kg/h IMD probably tended to cause deterioration of microcirculation and was therefore not used for further investigations.

## Discussion

In the current study, IMD enhanced endothelial barrier function in vitro and diminished lung hyperpermeability, but not pulmonary and systemic inflammation in a clinically relevant mouse model of VILI.

Impairment of endothelial barrier function is a hallmark of VILI and ARDS, leading to development of lung edema, surfactant dysfunction and deterioration of pulmonary gas exchange [6]. Particularly preinjured lungs are susceptible to VILI, and VILI may contribute to lung injury in ARDS patients despite lung protective ventilation strategies [4], [5]. A pharmacologic approach to reduce pulmonary permeability in addition to lung protective ventilation strategies thus may be a promising adjuvant therapeutic strategy to minimize VILI in ARDS patients.

IMD is an endogenous CRLR ligand that mediated endothelial barrier stabilizing effects in vitro and in an isolated lung model of hydrostatic lung edema [10], [11]. In mechanically ventilated mice, we investigated the regulation of IMD and its receptor complex composed of CRLR and one of the auxiliary proteins RAMP1, 2 and 3, which are mandatory for efficient CRLR signalling [8], [9]. Expression of IMD, CRLR and RAMP1 and 2 were not affected by MV, whereas RAMP3 expression was significantly reduced. Previous studies suggested that IMD predominantly signals through receptor complexes composed of CRLR and RAMP2 or 3 [8], [9]. Thus, the observed down regulation of RAMP3 may indicate reduced efficacy of endogenous IMD signalling in VILI, and treatment with exogenous IMD may restore or further amplify endogenous barrier stabilizing IMD effects in VILI.

Indeed, in the currently employed mouse model, continuously infused IMD markedly reduced lung permeability in VILI. To the best knowledge of the authors this study is the first providing evidence for pulmonary permeability reduction due to exogenous IMD in VILI. Mechanisms suggested to be responsible for the barrier stabilizing properties of CRLR signalling include increase of intracellular cAMP, leading to a protein kinase A dependent inhibition of myosin light chain phosphorylation and stabilization of intercellular junctions, finally leading to reduced endothelial intercellular gap formation [10], [11], [24], [25]. This mechanism, originally studied by CRLR stimulation with AM has been confirmed in a murine VILI model, in models of ALI and in isolated rat intestine exposed to bacterial toxin [7], [24], [26], [27].

Despite marked reduction of VILI-induced pulmonary permeability in IMD treated individuals, oxygenation was not improved. This contradictory finding may be partly explained by vasodilatory effects displayed by IMD. Specifically in the current study, increased pulmonary vascular resistance due to thromboxane receptor stimulation was reduced by IMD in isolated perfused and ventilated mouse lungs, and HPV in murine lung tissue slices was diminished by IMD. Although direct evidence is not provided by the current study, it is tempting to speculate that reduction of pulmonary vascular resistance by IMD resulted in ventilation/perfusion (V/Q) mismatch and increment of pulmonary shunt perfusion, probably masking improved oxygenation capacity due to reduced oedema formation in IMD treated individuals.

Pulmonary hyperinflammation is a central feature of VILI and contributes to lung permeability [6]. Previously, the CRLR ligand adrenomedullin ameliorated inflammatory responses in polymicrobial sepsis suggesting that CRLR signalling may mediate immunomodulating effects [28], [29]. In hyperoxic VILI, we observed ameliorated leukocyte infiltration into the alveolar space due to AM treatment [7], which was not evident in the current study using 0.025 mg/kg/h IMD. Also, cytokine production was unaltered by IMD. Notably in preliminary experiments, IMD in a higher dosage (0.075 mg/kg/h) reduced neutrophil counts in the BAL fluid of IMD treated mice subjected to VILI as compared to solvent treated controls by trend (p = 0.057; n = 4; data not shown in detail). Concurrently, neutrophil counts were significantly reduced in the blood and increased in lung tissue (p<0.05; n = 4; data not shown in detail), probably as a result of leukocyte sequestration due to deterioration of microcirculation, as lactate levels were elevated in these mice. In line, IMD has been reported to lower systemic blood pressure in rodents [8], [13], [30]. In preliminary experiments (n = 4), 0.075 mg/kg/h IMD lowered mean arterial blood pressure and compromised systemic microcirculation. However, treatment with 0.025 mg/kg/h IMD reduced MAP only moderately and did not result in compromised systemic microcirculation or relevant reduction of end organ perfusion pressure as indicated by comparable blood lactate levels and urine output in both groups. Thus, IMD most likely reduced endothelial gap formation in VILI by inflammation-independent mechanisms in the current study, which may hold relevant clinical benefits. First, in ventilated ARDS patients with sepsis or severe pneumonia, hyperinflammation may override anti-inflammatory properties of pharmacologic therapies. Second, limiting central mechanisms of the innate immune response may compromise host defence against invading pathogens and thereby pave the way for secondary bacterial infections.

In conclusion, the endogenous CRLR ligand peptide IMD reduced pulmonary vascular leakage in a clinically relevant mouse model of VILI. Reduction of lung permeability was not accompanied by reduced pulmonary or systemic inflammation. However, regarding oxygenation capacity IMD may counteract reduction of lung edema by increasing V/Q mismatch.

## Supporting Information

Figure S1 **Preabsorption of the IMD antibody with mouse IMD (1–47) resulted in almost complete absence of labelling, suggesting high specificity of the primary antibody.** Both tissue sections were cut from the same specimen and processed simultaneously. Images were taken at the same exposure time (190 ms).
(TIF)

Figure S2 **Double labelling with LEA to depict the epithelial lining of the alveolus in non ventilated lungs.** The merged image also includes DNA labelling with DAPI to highlight cellular nuclei. There is little overlap of LEA binding to alveolar type I cells with IMD- or CRLR-immunoreactivity. *Arrows* in the merged image point to LEA-binding epithelial stretches without IMD- or CRLR-immunoreactivity.
(TIF)

Figure S3 Mice were ventilated with a tidal volume of 12 ml/kg for 6 h and treated with IMD 0.025 mg/kg*h (6 h vent.+IMD) or solvent (6 h vent). A) Mean arterial blood pressure (MAP) was monitored. MAP was lower in IMD treated individuals B) Lactate levels quantified at the end of the experiment and C) urine output were not different between groups (***p<0.001; n = 15).
(TIF)

Table S1 (DOC)
