# Supplementary figures and images for: Intermedin Stabilized Endothelial Barrier Function and Attenuated Ventilator-induced Lung Injury in Mice
Source: PLoS One. 2012 May 1;7(5):e35832. doi: 10.1371/journal.pone.0035832 (PMC3341380; doi:10.1371/journal.pone.0035832)

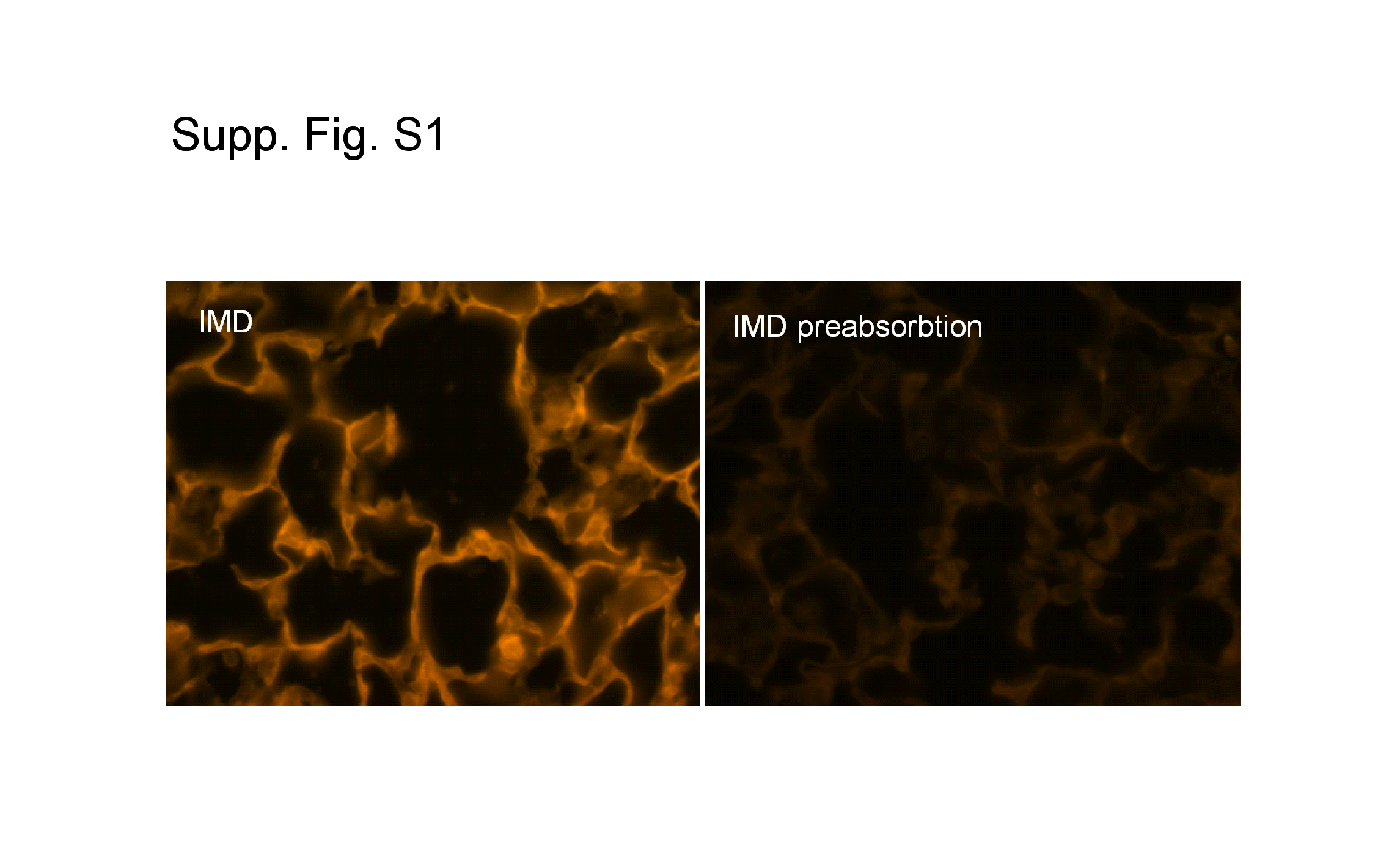

Supplement: Figure S1 — Preabsorption of the IMD antibody with mouse IMD (1–47) resulted in almost complete absence of labelling, suggesting high specificity of the primary antibody. Both tissue sections were cut from the same specimen and processed simultaneously. Images were taken at the same exposure time (190 ms). (TIF) [file pone.0035832.s001.tif]

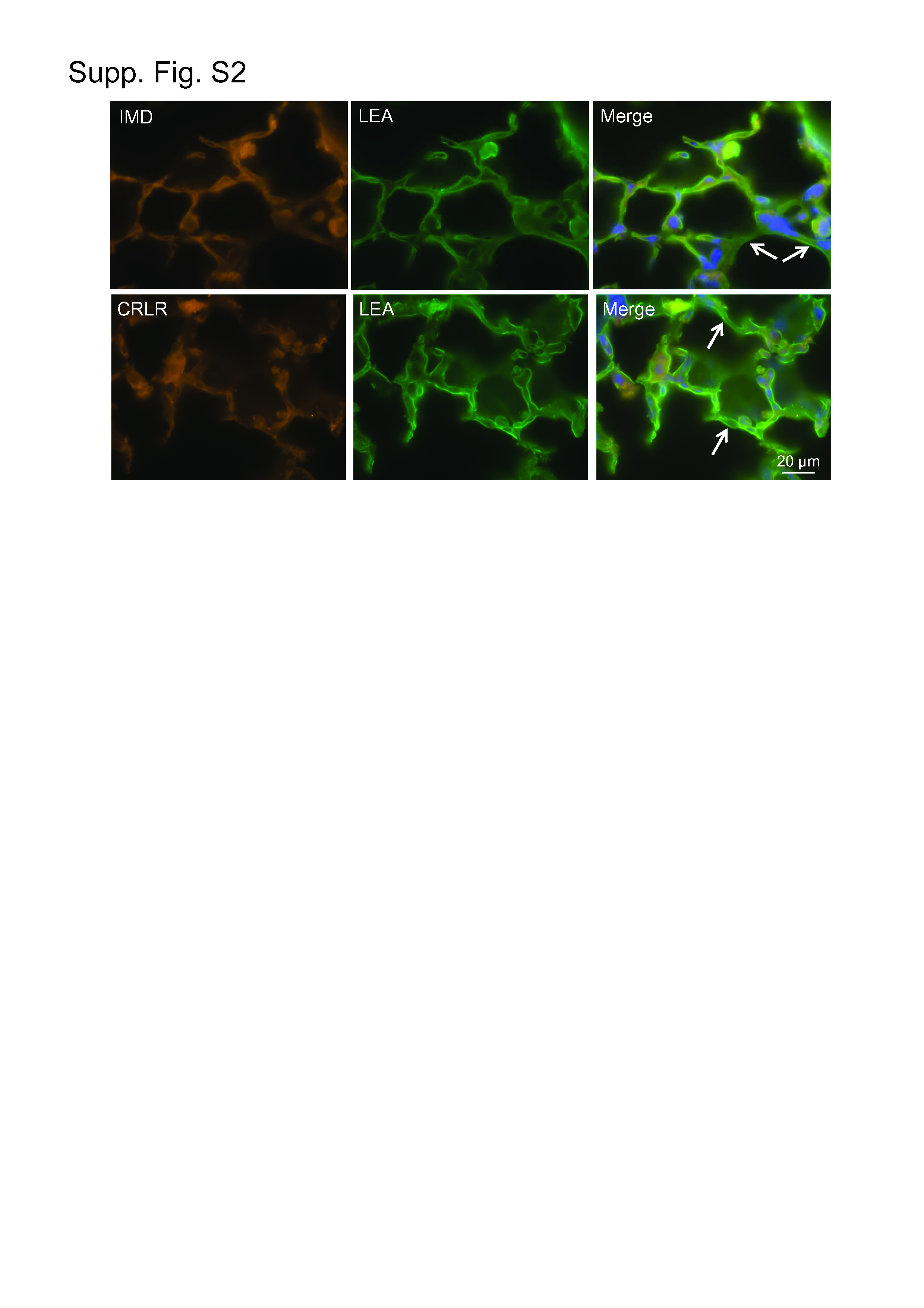

Supplement: Figure S2 — Double labelling with LEA to depict the epithelial lining of the alveolus in non ventilated lungs. The merged image also includes DNA labelling with DAPI to highlight cellular nuclei. There is little overlap of LEA binding to alveolar type I cells with IMD- or CRLR-immunoreactivity. Arrows in the merged image point to LEA-binding epithelial stretches without IMD- or CRLR-immunoreactivity. (TIF) [file pone.0035832.s002.tif]

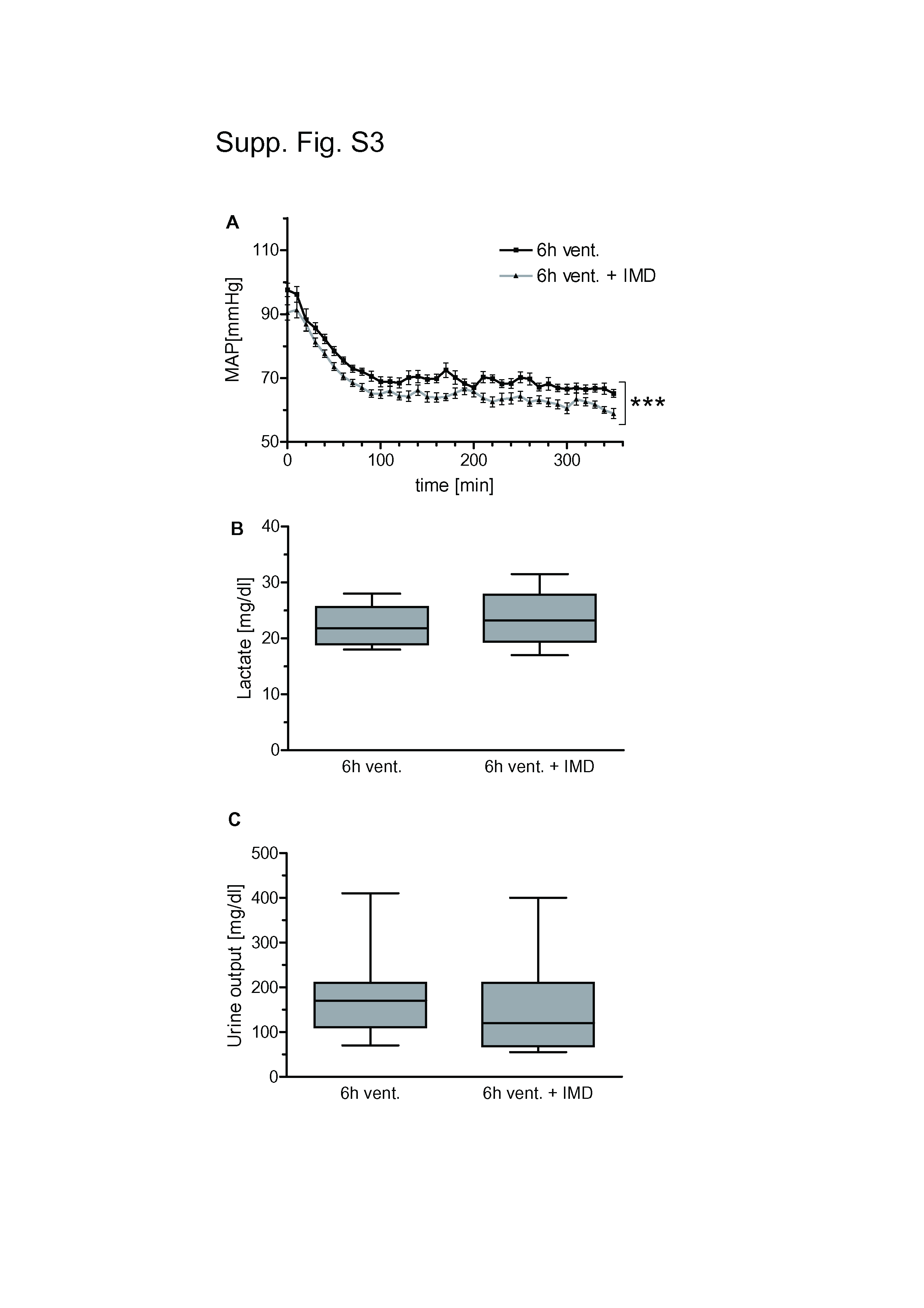

Supplement: Figure S3 — Mice were ventilated with a tidal volume of 12 ml/kg for 6 h and treated with IMD 0.025 mg/kg*h (6 h vent.+IMD) or solvent (6 h vent). A) Mean arterial blood pressure (MAP) was monitored. MAP was lower in IMD treated individuals B) Lactate levels quantified at the end of the experiment and C) urine output were not different between groups (***p<0.001; n = 15). (TIF) [file pone.0035832.s003.tif]
