# Supplementary material for: Intermedin Stabilized Endothelial Barrier Function and Attenuated Ventilator-induced Lung Injury in Mice
Source: PLoS One. 2012 May 1;7(5):e35832. doi: 10.1371/journal.pone.0035832 (PMC3341380; doi:10.1371/journal.pone.0035832)
Supplement: Table S1 — (DOC) [file pone.0035832.s004.doc]

Supplemental Table 1

Primer used for RT-PCR

---------------------------------------------------------------------------------------------------------------

gene sequence product accession

length number

---------------------------------------------------------------------------------------------------------------

IMD forward GGTAACCCTCGGTTGCATCAG 178 bp NM182828

reverse GGCATGACGACGAGACTTCC (197-375)

β-actin forward GTGGGAATGGGTCAGAAGG 299 bp NM007393

reverse GGCATACAGGGACAGCACA (212-511)

CRLR forward GCAGGACCCCATTCAACA 185 bp AF209905

reverse GGATGCCGAAACCAGTGT (169-345)

CGRP forward CTCCCCTTTCCTGGTTGT 201 bp NM007587

reverse TCAGCCTCCTGCTCTTCCT (173-374)

RAMP1 forward ATGGTGTGACTGGGGAAAGA 205 bp NM031645

reverse CAATGAAAGGGCAGAGGATG (205-410)

RAMP2 forward TCCCTGAACCAATCTCTTCC 185 bp NM019444

reverse GTCGCTGTAATGCCTGCTAA (260-445)

RAMP3 forward GCAACGAGACAGGGATGC 312 bp BC024765

reverse GCCACAGTCAGCACGACA (116-428)

----------------------------------------------------------------------------------------------------------------
